# Providing colored photoperiodic light stimulation during incubation: 1. Effects on embryo development and hatching performance in broiler hatching eggs

**DOI:** 10.1016/j.psj.2021.101336

**Published:** 2021-06-19

**Authors:** Xujie Li, Bruce Rathgeber, Nancy McLean, Janice MacIsaac

**Affiliations:** ⁎ Department of Animal Science and Aquaculture, Faculty of Agriculture, Dalhousie University, Truro, NS B2N 5E3, Canada; † Department of Plant, Food, and Environmental Sciences, Faculty of Agriculture, Dalhousie University, Truro, NS B2N 5E3, Canada

**Keywords:** photostimulation, light color, air cell temperature, hatchability, chick quality

## Abstract

Providing lighting schedule during incubation has been shown to improve chick quality and reduce stress posthatch. This study was conducted to evaluate the effects of providing light of different colors during incubation on embryo development, air cell temperature, the spread of hatch, and hatching performance. Four batches of eggs (n = 2,176, 1,664, 1,696 and 1,600) from Ross 308 broiler breeders were used in the experiment. In each trial, eggs were randomly distributed into 4 lighting treatments. The incubation lighting treatments included: incubated under dark as control, illuminated with white, red or blue lights for 12 h daily. There were no incubation lighting treatment differences in embryo development, the spread of hatch, hatchability, embryo mortality, hatch weight, chick length, navel closure quality, yolk-free body weight, or relative spleen weight. However, embryos incubated under red light had lower average air cell temperature than those in dark, white or blue light treatments. This finding may suggest higher melatonin secretion during the scotophase when illuminated with red light. Male chicks incubated under dark had a higher bursa of Fabricius weight than males illuminated with blue light. In conclusion, these results suggest that the red, white and blue light stimulation during incubation had no negative effects on hatchability, embryo mortality, spread of hatch or day-old chick quality, but may have potential impacts on immunity and energy metabolism in broiler embryos.

## INTRODUCTION

Avian embryogenesis is a perfect platform to examine the effects of exogenous factors on embryonic development due to the physiological independence from the hen (Hill et al., 2004). During incubation, the management of cabinet temperature, humidity, egg turning and air composition are critical to achieving successful artificial incubation. Light is an important exogenous factor for controlling many physiological and behavioral processes in animals but the use of light in artificial incubation is not commonly practiced. In nature, hens are off the nest for drinking and eating, especially during the last week of incubation (Archer and Mench, 2014b). The developing chicken embryos would receive light stimulation when hens were off-nest. Normally, commercial incubation units are not illuminated except for when humans enter to deliver eggs or to clean the unit after use.

We know, from in vitro studies, that the pineal gland of avian embryos responds to light exposure. Aige-Gil and Murillo-Ferrol (1992) reported a significant increase in the number and size of pineal intracytoplasmic lipid droplets in embryos exposed to light for 18 d of incubation. Light sensing opsins can be detected in an embryo on d 14 of incubation (Bruhn and Cepko, 1996).

Light intensity, the composition of the spectrum and photoperiod (daily pattern of light and dark exposure) are 3 main parameters of light when used as a tool to manage poultry production. There has been interest in determining the impact of providing light to the incubation environment on hatching performance and the quality of newly hatched chicks. Implementing a light regime during incubation has been studied for over 50 yr (Peters et al., 1958). Incandescent and fluorescent lamps were used in early studies and had a positive effect on accelerated embryo development and a decrease in incubation time to hatch (Erwin et al., 1971; Andrews and Zimmerman, 1990; Shafey and Al-mohsen, 2002; Archer et al., 2009; Cooper et al., 2011). But the additional heat emitted from the light source may be confounded with the effects of light (Rozenboim et al., 2003). Recently, the light-emitting diode (**LED**) lamp has become commercially available and produces far less heat than conventional lamps. Since then, numerous studies have been conducted to examine the effects of application of LED lights during incubation on embryo development and hatching performance parameters (Rozenboim et al., 2004a; Özkan et al., 2012; Archer and Mench, 2013; Archer, 2017; Archer et al., 2017; Sabuncuoğlu et al., 2018; Hannah et al., 2020).

It has been found that light stimulation during incubation could potentially affect embryonic cell proliferation measured by changes in total embryo and breast muscle weight (Rozenboim et al., 2004a; Halevy et al., 2006). Archer and Mench (2014a) found a rhythmic secretion of melatonin on d 19 of incubation when illuminated with photoperiodic light during embryogenesis. Eggs incubated in the presence of light have been shown to have accelerated embryo development (Shafey and Al-mohsen, 2002; Cooper et al., 2011), increased hatchability (Shafey and Al-mohsen, 2002; Archer, 2017; Archer et al., 2017), and improvements in the quality of chicks at hatch (Archer, 2017; Archer et al., 2017). However, the results on embryonic development, hatchability and chick quality are inconsistent. Some studies demonstrated that light stimulation during incubation reduce or do not affect hatchability or body weight at hatch (Archer et al., 2009; Özkan et al, 2012; Archer and Mench, 2014a; Zhang et al., 2016; Sabuncuoglu et al., 2018). These disagreements among studies suggest that there are some factors influencing the response of chicken embryos to light stimulation during incubation. Melatonin is produced rhythmically when vertebrates are entrained by the day and night cycle, and it is involved in thermoregulation, metabolic functions and immune responses in chickens (Zeman et al., 2001; Gharib et al., 2008). Whether embryonic growth rate and the development of the skeletal system are affected by rhythmic melatonin production during embryogenesis is not yet clear.

Hatchlings are removed from incubators when the majority of chicks have hatched. The duration from the time of first chick to emerge to late-hatching chicks can be up to 48 h (Decuypere et al., 2001). This time period is commonly referred to as the hatch window (**HW**) (Decuypere et al., 2001). An extended HW has been reported to be associated with poor chick quality at hatch, which negatively affects the welfare and growth performance of a flock after placement at a rearing facility. Improved synchronization of hatch can decrease the number of dehydrated chicks. Hannah et al. (2020) found that Lohmann Brown embryos took less time to reach 50% of total chicks hatched and reduced the time between 50% and 75% of total chicks hatched when 12 h d^−1^ of full spectrum light was provided for the entire incubation period. This indicates that there is potential for improved hatch synchronization with the use of lights. There are few studies regarding the effect of light spectra on hatch time. Rozenboim et al. (2004a) reported no difference in incubation time for eggs incubated in the dark compared to eggs illuminated with monochromatic green light (15 min L: 15 min D) for broiler chicken embryos. Zhang et al. (2016) found that continuous illumination with green light did not affect hatch time or HW for broilers. However, an earlier study found that embryos incubated under 20-watt green fluorescent light required less time to hatch than those incubated in the dark (Shafey and Al-mohsen, 2002). Furthermore, it is found that longer light exposure required less time to hatch in broilers (Walter and Voitle, 1972). The shortened incubation time from light exposure that may be simply due to an overheating effect by conventional light source or related to an accelerated embryonic growth rate or higher embryo activity. The mechanism involved in this process during incubation is still unknown.

There is evidence from studies with broiler hatching eggs exposed to different wavelengths of light that wavelength is influential in embryo response. Archer (2017) reported an improvement in hatchability and lower susceptibility to stress when broiler eggs were given 12 h d^−1^ of white or red LED lights as compared to green light or a dark environment. It makes sense that embryos may respond differently to different wavelengths of light based on what we know from studies posthatch. Light wavelengths can affect the growth, development and behavior of chickens during rearing (Rozenboim et al., 2004b; Xie et al., 2008; Sultana et al., 2013). Sensitivity of birds to specific wavelengths of light varies at different developmental stages including during incubation and posthatch. Green light has been reported to accelerate broiler growth at an early age, while blue light had stronger effects on growth of broilers after 10 d of age (Rozenboim et al., 2004b). The color of the eggshell also affects how the light passes through and is received by embryos. This may explain why researchers like Hannah et al. (2020) found differences in distribution of hatch among lines of chickens that lay different colored eggs. However, few conclusions can be drawn regarding the optimal wavelength and its combination with photoperiod on embryo development and hatching performance parameters. Information on effects of entrainment of circadian rhythm by different colors of light for chicken embryo during incubation period on embryonic development, the spread of hatch, and day-old chick quality is limited. The objective of this project was to evaluate the effects of providing white, blue or red LED lights for 12 h daily during incubation period on embryonic development, embryo mortality, hatchability, air cell temperature, the spread of hatch and chick quality in commercial broiler chickens. We hypothesized that different colors of photoperiodic LED light exposure during incubation would affect the early entrainment of circadian rhythm and result in different responses for embryo growth and hatching traits.

## MATERIALS AND METHODS

The experimental protocol was carried out in accordance with the Canadian Council of Animal Care Guidelines (CCAC, 2009).

### Incubation

**Figure 1:**
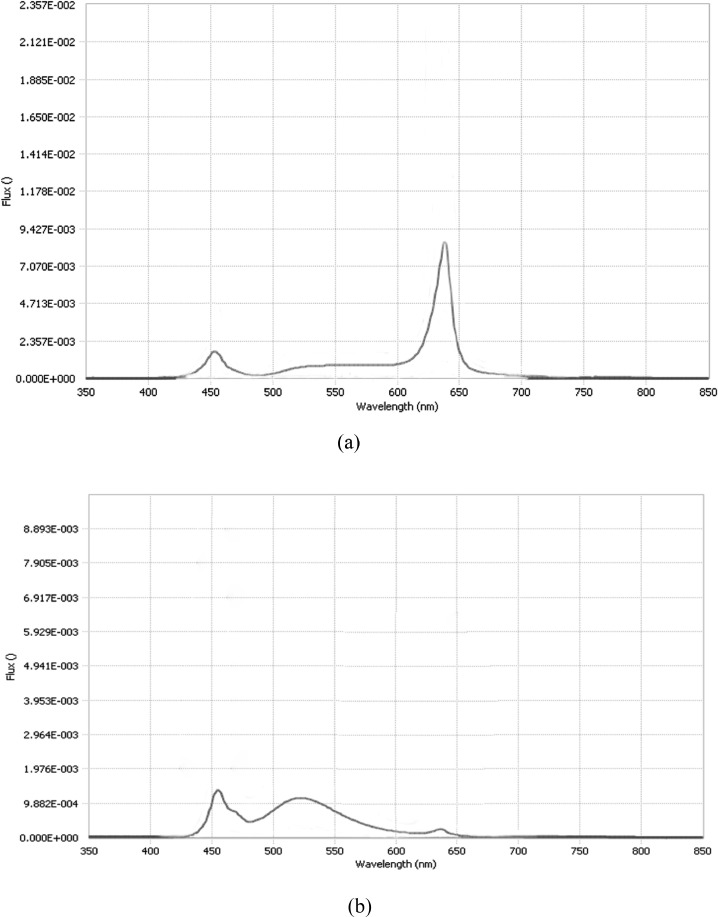
Spectrum of red (A) LED light dimmed at 60% and blue (B) LED light dimmed at 40% at egg level within incubator.

The experiment consisted of 4 incubation trials. In the first trial, a total of 2,176 hatching eggs from a 54-wk Ross 308 broiler breeder flock were obtained from a commercial hatchery. Eggs were randomly divided into 4 experimental groups with 2 replicate incubators. A total of 8 recently calibrated ChickMaster single-stage incubators (ChickMaster G09, Cresskill, NJ) with a capacity of 1,188 eggs per incubator were used for each trial. Two incubators were operated without light (0L:24L, Dark) as a control group, the remaining 6 incubators were illuminated with white (color temperature 4100K, Canarm, Brockville, ON, Canada), red (Once Innovations, Plymouth, MN) or blue (Once Innovations, Plymouth, MN) LED lights from the beginning of incubation. The spectrum of red and blue light is presented in Figure 1. Four LED strips were attached to metal frames on the left side within each incubator and were on 12 h d^−1^ (0700 to 1900 h). Light intensity at egg level was adjusted to 200 lx, as measured with a digital luxmeter (61-686 Digital Light Meter, Ideal, Ajax, Canada). The LED lights were low profile and did not restrict airflow within the incubator. Eggs were turned through 90° once every 45 min for the first 444 h (18.5 d) of incubation. Incubation units were maintained at a temperature of 37.5°C for the entire incubation period. During the first 476 h (19 d plus 20 h) of incubation, relative humidity (**RH**) was set at 55%. RH was increased to 64% at 476 h, 72% at 488 h, 82% at 509 h and set back to 55% at 509 h of incubation (3 h before pulling out). The temperature, humidity and light intensity were monitored using data loggers (OMYL-M62, Omega Engineering, St-Eustache, QC, Canada) placed in the incubators to ensure that conditions were comparable among incubators. All eggs were candled at 444 h of incubation. Nonviable eggs were removed and broken open to determine fertility status. If fertile, the day of embryonic death was estimated and categorized as early (d 0–7), middle (d 8–14), or late (after d 15) death. The viable eggs were transferred into hatching trays and incubated in the original incubators without turning for the remaining incubation period.

The same experimental design was applied in all trials. The second, third and fourth trials had 1,664, 1,696 and 1,600 eggs from breeders at 31, 55 and 38 wk of age, respectively. The lighting treatments were rotated among incubators for each trial so that each treatment was represented once in each incubator to prevent the systematic effect of the incubation units.

### Embryonic Development

Embryo development parameters were measured at 1 h after lights came on (0800 h) on embryonic d (**ED**) 10, 14, and 18. An additional 192 eggs within a narrow weight range were weighed and randomly distributed into 8 incubators to evaluate embryonic development. On each of ED 10, 14 and 17 of incubation, 8 numbered eggs per incubator were removed from the incubator and weighed individually. The percentage of egg weight loss was calculated using the initial egg weight. Eggs were opened and the front-to-back eyeball diameters of both eyes and embryo diameter were measured by digital calipers from the outermost portion of the curled tail to the outermost part of the curved neck with the natural curled position. Then embryos were separated from the residual yolk and euthanized by cervical dislocation. The residual yolk weight (only on ED 14 and 18), embryo length and weight, diameter and weight for both eyes, and heart weight were measured to the nearest 0.1 mm and 0.001 g, respectively.

### Air Cell Temperature

Eighty eggs (10 eggs per incubator) within a narrow weight range were set on the top tray of each incubator to measure the embryo temperature twice per d. Air cell temperature was measured with transponders (implantable programmable temperature transponder, IPTT-300; BMDS Inc., DE, USA) in the trial 1, 3 and 4. All transponders were factory-calibrated for a range of 32°C to 43°C with an accuracy of 0.5°C and a resolution of 0.2°C before arrival. Each transponder was sterile and contained a battery-free microchip, read with a probe (DAS-6007 Smart probe, BMDS Inc., DE, USA). The probe reader detected a low frequency radio signal from the transponder within a range of 5 cm and received transponder ID and temperature. On ED 7.5, the preselected eggs were candled and implanted with transponders in the air cell of eggs with viable embryos. The eggshell surface area surrounding the site of eggshell perforation was disinfected with 70% ethanol. An oblong shape opening (around 3 mm width and 1 cm length) was perforated in the large end of each egg. Subsequently, a sterile transponder was inserted into the air cell without disrupting the inner shell membrane and chorioallantoic membranes. The opening was covered with a piece of clear packing tape and sealed with melted paraffin wax. Egg weight was determined before and after implantation, and on ED 18. Between ED 8 and ED 18, air cell temperature was measured every 12 h (1 h after lights turned on or off). In the current study, approximately 74% (178 out of 240) of eggs implanted with transponders in the air cell were hatched and mortality did not differ (*P* > 0.05) among incubation lighting treatments.

### Spread of Hatch

The number of hatched chicks per tray was recorded in 3 h intervals from 454 h to 512 h of incubation to calculate the spread of hatch. Chicks were considered as hatched when they were completely emerged and free from the eggshell. The counting process was carried out in the dark by using a head lamp (0.1–0.2 lux) as the only source of light to minimize the effects of external light on the embryos. At each check, the total number of chicks hatched within the 3 h period was moved to the back of each tray (separated by a barrier) and recorded.

### Hatchability and Chick Quality

At the end of incubation (512 h of incubation), all hatched chicks were counted and weighed in a batch for each incubator. Hatchability was calculated based on total fertile eggs. The chicks were feather sexed and chick quality was assessed by navel closure condition and overall appearance. Navel closure condition was scored as 1 (clean and closed navel area), 2 (black button up to 2 mm or black string), or 3 (black button exceeding 2 mm or open navel area) (Molenaar et al., 2010). In addition, the chick length of 40 males and 40 females per incubator/replicate were randomly selected and measured. All unhatched eggs were broken open to determine the approximate d of embryonic death. An additional 8 male and 8 female chicks per replicate were randomly selected and euthanized by CO for assessing chick development. The intact chick was weighed and dissected. The residual yolk and yolk-free body weight (**YFBW**) were calculated as percentage of intact chick weight. Spleen, bursa of Fabricius and liver were harvested and calculated as the percentage of YFBW.

### Statistical Analysis

This experiment was a randomized complete block design with a set of 4 incubators as one block (2 blocks per trial). Incubator was used as the experimental unit with 8 blocks for each lighting treatment. One incubator (dark-control) in trial 2 was not functioning as consistently as the other 7 incubators and was removed from the analysis. The residuals of error met the assumptions of normal distribution, independently distributed with mean zero and had constant variance before further analysis. Embryonic development data were analyzed by sampling day using the Mixed Procedure of SAS v. 9.4 (SAS Inc., Cary, NC, 2013). The air cell temperature data were analyzed as repeated measures in SAS v. 9.4 (SAS Inc., Cary, NC, 2013). In repeated measures analysis, 4 covariance structures, first order autoregressive, compound symmetry, toeplitz, and variance components were compared. The covariance structure which provided the lowest absolute values for Akaike Information Criterion and Bayesian Information Criterion was selected for repeated measures analysis. Based on these criteria, covariance structure toeplitz was selected for all parameters. The spread of hatch data was analyzed using nonlinear regression model of SAS v. 9.4 (SAS Inc., Cary, NC, 2013). A 3-parameter logistic growth model (Eq. 1) was found to be the best nonlinear regression model that described the relationship between the cumulative percentage of hatched chicks (Y) and h of incubation (X) for all treatments. To compare the spread of hatch among lighting treatments, a new equation (Eq. 2) was converted from the predicted model (Eq. 1) for each experimental unit (incubator). The h of incubation reaching 10%, 25%, 50%, 75% and 90% of chicks hatch were calculated and then analyzed using the Mixed Procedure of SAS v. 9.4 (SAS Inc., Cary, NC, 2013).

(1) $$Y=\frac{\theta_{1}}{1+\exp(-\frac{\left(X-\theta_{2}\right)}{\theta_{3}})}+\varepsilon$$

(2) $$X=\theta_{2}-\left[\ln\left(\frac{\theta_{1}}{Y}-1\right)\theta_{3}\right]+\varepsilon$$

*The parameters represent the asymptote (*θ_1_*), the time to half the asymptote (*θ_2_*), and the interval from half to until ¾ of the asymptote (*θ_3_*).

Data for hatching performance (initial egg weight, fertility rate, hatchability, egg weight loss percentage, embryo mortality, and hatch weight) were analyzed using the Mixed Procedure of the SAS v.9.4 (SAS Inc., Cary, NC, 2013) to determine the effect of lighting treatments. For the navel score, chick length and chick quality parameter obtained from dissected chicks, the statistical model included the gender effect. In all cases, if significant effects were found, the Tukey-Kramer test was applied to differentiate the means at 5% level of significance.

## RESULTS AND DISCUSSION

### Embryo Development

There were no differences (*P* > 0.05) among the 4 groups regarding percent of weight loss, embryo size and weight, residual yolk weight, eyeball diameter, or relative heart weight on either ED 10, 14, or 18 (Table 1). Similar results have been reported previously (Zhang et al., 2016; Dishon et al., 2017). Dishon et al. (2017) reported that illuminated broiler eggs with LED light did not affect body, muscle, or liver weight. However, the effects of the photostimulation on embryo development regarding the embryo weight or physiology parameters were not consistent in past studies. Coleman and McDaniel (1976) reported that White Leghorn embryo development was accelerated from the first d of receiving continuous white fluorescent light. Similar results have been found in Northern Bobwhite quail (Walter and Voitle, 1973); House sparrows (Cooper et al., 2011), Hybro broilers (Shafey and Al-mohsen, 2002), and layers (Shafey, 2004) when illuminated with incandescent or fluorescent light during incubation. The additional heat from those conventional lights may have an overheating effect and have a cofounding effect with light stimulation on the embryo development. Wang et al. (2014) reported advanced embryo development with continuous green LED light exposure during incubation for chicken embryos. However, no differences in embryo development or liver index at ED 15 and 18 among red, blue and dark were found in their study, which indicates that light wavelength may be a critical factor affecting embryo and organ development. Rozenboim et al. (2004a) reported higher embryo weight as percent of egg weight on ED 14, 15, 17 and 20 but not at hatching when illuminating with monochromatic green LED light (15 min ON and 15 min OFF). The mechanism behind acceleration of embryo development may be related to an alteration of somatotropic axis hormones (growth hormone, insulin-like growth factor-I, and prolactin) production from light exposure. A significant increase in hypothalamic growth hormone-releasing hormone RNA expression was found from ED 16 to ED 20 when embryos were illuminated with green light (15 min light/15 min dark). In addition, plasma growth hormone levels were significantly higher on ED 14, 16, 18, and 20 in embryos with green light exposure (Dishon et al., 2017; 2018).

**Table 1:** Effects of the application of colored photoperiodic light during incubation on egg weight loss (EWL) (%), the weights of embryo (% of egg weight) and organs (g kg^−1^), embryo length (mm), the diameter of embryo (mm) and eyeballs (mm), and the relative embryo diameter (mm g^−1^) and length (mm g^−1^) at embryonic d (ED) 10, 14 and 18 of incubation in Ross 308 broiler embryos.

Item	n^1^	EWL	Relative embryo weight^2^	Yolk weight^3^	Embryo diameter	Relative embryo diameter^4^	Embryo length	Relative length^5^	Eyeball diameter	Relative heart weight^6^
Variation source (ED10)										

Dark	7	6.53	4.50	-	31.0	10.76	-	-	8.2	8.36
Blue	8	7.07	4.41	-	30.8	10.99	-	-	8.2	8.03
Red	8	6.90	4.44	-	30.7	10.95	-	-	8.1	8.35
White	8	6.54	4.39	-	30.8	10.93	-	-	8.1	8.36
SEM		0.211	0.048	-	0.31	0.149	-	-	0.05	0.157

Lighting treatment		0.237	0.443	-	0.865	0.745	-	-	0.372	0.379
Block		0.686	<0.0001	-	0.0001	<0.0001	-	-	<0.0001	0.001

Variation source (ED14)										

Dark	7	9.38	22.14	18.6	53.9	3.80	104.8	7.4	10.9	7.62
Blue	8	8.92	22.53	18.6	54.9	3.78	103.5	7.1	10.9	7.67
Red	8	9.51	22.26	18.5	53.9	3.79	103.0	7.2	10.8	7.76
White	8	9.45	22.39	18.6	54.6	3.78	103.5	7.2	11.0	7.62
SEM		0.220	0.314	0.20	0.87	0.070	0.75	0.10	0.07	0.108

Lighting treatment		0.248	0.847	0.986	0.816	0.994	0.457	0.319	0.590	0.753
Block		0.001	<0.0001	<0.0001	0.901	0.072	<0.0001	<0.0001	0.003	0.002

Variation source (ED18)										

Dark	7	11.86	46.69	13.4	56.7	1.89	159.1	5.3	11.9	7.29
Blue	8	11.81	46.57	13.6	56.8	1.90	157.4	5.2	11.9	7.49
Red	8	12.23	46.19	13.3	57.1	1.92	158.8	5.3	11.9	7.47
White	8	11.69	46.69	13.3	57.3	1.91	159.3	5.3	11.9	7.45
SEM		0.300	0.270	0.25	0.54	0.02	0.77	0.03	0.06	0.084

Lighting treatment		0.626	0.537	0.782	0.886	0.861	0.320	0.309	0.992	0.415
Block		0.012	<0.0001	<0.0001	0.037	<0.0001	<0.0001	<0.0001	0.0002	0.002

1 Number of experimental units. Experimental unit = 8 eggs per incubator per sampling day.

2 Relative embryo weight = Embryo weight/egg weight x 100%.

3 Yolk = Yolk weight/day 0 egg weight x 100%.

4 Relative diameter to embryo weight = Embryo diameter/embryo weight x 100%.

5 Relative length to embryo weight = Embryo length/embryo weight x 100%.

6 Relative heart weight = Heart weight/embryo weight x 100%.

Accelerated embryo development as a result of light exposure was not detected in the current study. Several factors that characterize light that can affect embryo development need to be considered. These factors include the source of the light, the duration of light exposure, the spectra produced and the intensity of light. Acceleration of embryonic development was found in some studies, which used different photoperiods during incubation. For instance, 15 min on and 15 min off (Rozenboim et al., 2004a), 12 h d^−1^ (Walter and Voitle, 1972; 1973) and 16 h d^−1^ of light exposure (Özkan et al., 2012; Yu et al., 2018) had positive impacts on embryo development. Those results suggested that providing photoperiod during incubation may stimulate pineal melatonin secretion and regulate growth hormone synthesis. However, an increased overall embryo weight as well as embryo muscle weight were also found when light exposure was continuous, which excluded the exogenous time Zeitgeber for chicken embryos (Shafey and Al-mohsen, 2002; Wang et al., 2014). Those results indicated that muscle growth may depend on light exposure, but was not only associated with circadian rhythms entrained by photoperiod. Light wavelength and intensity can influence the amount of light that can pass through the eggshell and reach the embryo. The light intensity in the current study may be one of the key factors affecting mitosis in neural crest mesoderm during the early stage of embryo development. Yu et al. (2018) found green LED light at a low intensity (50 lux) stimulated embryo growth during incubation. However, no improvement in embryo weight to initial egg weight was detected when green light was set to 150 or 300 lux. The intensity in our study was approximately 200 lux at egg level, which may be too high as reported by Yu et al. (2018). However, an increased embryo weight was found when illuminated with fluorescent light at 200 to 300 lux (Özkan et al., 2012). Whether light intensity of LED light at 200 lux stresses the broiler embryos should be evaluated in a future study. The difference between findings on embryo weight may relate to the light source, wavelength, intensity, or their combination with eggshell characteristics, such as, pigment intensity ( Shafey et al., 2004) and thickness (Maurer et al., 2015). The response to incubation light stimulation may also vary among avian species. Further studies are needed to investigate whether genetic factors are closely related to cell proliferation, hormone (growth hormone and IGF-1) regulation and embryo development when exposed to photostimulation during incubation.

Eyeball diameter and heart weight were not affected by the presence of light during incubation in the current study (Table 1). Zhang et al. (2016), who reported similar results, incubated eggs with continuous green or white light and did not find differences in relative weight of heart, liver and eyeball among treatments. The dimensions of the chicken eyeball at market age are not affected by the provision of fluorescent light (550 lux) during incubation (Archer et al., 2009). It has been reported that domestic chickens reared under continuous fluorescent light (1,044 lux) develop abnormal buphthalmic eyes (Whitley et al., 1984). Our results indicated that providing LED light at 200 lux up to 12 h daily did not negatively impact eye development.

### Air Cell Temperature

The air cell temperature was significantly affected by light color treatments (*P* < 0.01) as well as the embryo age (*P* < 0.01) (Table 2). Embryos incubated under red light with 12 h d^−1^ of light exposure had lower air cell temperature than those in the dark, or with white or blue light. There were no differences in air cell temperature among white, blue and dark groups. Archer et al. (2017) reported that eggshell temperature was not affected by illumination with red LED light. Considering the results of air cell temperature and egg weight loss percentage (Tables 1 and 2), providing LED light at 200 lux for 12 h d^−1^ does not cause an over-heating effect on broiler hatching eggs. Although, we do not have a clear explanation for lower air cell temperature found in eggs illuminated with red LED light for 12 h d^−1^. One possible explanation may be that red light increases the accumulative melatonin secretion during scotophase. Melatonin can interact with growth hormone production (Zeman et al., 1999) and thermoregulation (Rozenboim et al., 1998). Zeman et al. (2001) reported that reduced heat production in female broilers when providing 150 mg kg^−1^ of melatonin as a feed supplement at 2 and 3 wk of age. To the best of our knowledge, there are no previous studies reporting thermoregulation as an indicator for the establishment of a circadian rhythm for chicken embryos. It is worth monitoring air cell temperature continuously and its correlation to the melatonin production within a 24 h cycle in future research.

**Table 2:** Effects of the application of colored photoperiodic light during incubation on egg weight loss (%) and air cell temperature (°C) from d 7 to 18 of incubation in Ross 308 broiler embryos.

Wavelength	n^1^	Egg weight loss 0-18	Egg weight loss 7-18	Air cell temperature
Dark	5	12.34	7.38	37.8^a^
Blue	6	11.12	6.43	37.8^a^
Red	6	12.04	7.08	37.7^b^
White	6	12.23	7.20	37.8^a^
SEM		0.549	0.396	0.01

ANOVA *P*-value				

Wavelength (W)		0.411	0.382	<0.0001
Embryo Age (A)		N/A	N/A	<0.0001
W x A		N/A	N/A	1.000
Block		0.333	0.288	<0.0001

1 Number of experimental units. Experimental unit = 10 eggs per incubator in trial 1, 3, and 4.

a,b Means within column with different letters differ significantly according to Tukey-Kramer test (α = 0.05).

### Spread of Hatch

There were no differences in incubation time to reach 5%, 10%, 25%, 50%, 75%, 90% or 95% of chicks hatched among light color treatments (Table 3). In addition, the duration between specific percent of hatch did not differ among treatments (Table 4). Our results contrast with some previous studies (Walter and Voitle, 1972; Fairchild and Christensen, 2000; Shafey and Al-mohsen, 2002) which reported a reduction in hatch time by the presence of light during incubation, but agrees with the several studies, which reported no differences in time of hatch when providing light exposure during incubation for Japanese quail (Sabuncuoglu et al., 2018) and broilers (Rozenboim et al., 2004a; Özkan et al., 2012; Zhang et al., 2016). No differences in internal pipping time and length of hatch window were found between incubation in the dark and providing photoperiodic green light during the first 18 d of incubation in Ross broilers (Tong et al., 2018).

**Table 3:** Effects of the application of colored photoperiodic light during incubation on time in hours to reach a specific percentage of hatch (set time to hatch time) in total hatched Ross 308 broilers.

		% of chicks hatch						
Wavelength	n^1^	5%	10%	25%	50%	75%	90%	95%
Dark	7	483.2	486.4	491.0	495.6	500.0	504.2	506.8
Blue	8	480.8	484.3	489.6	494.8	499.8	504.4	507.0
Red	8	482.5	485.9	490.9	495.9	500.6	504.9	507.3
White	8	482.2	485.4	490.3	495.0	499.7	504.2	506.9
SEM		1.19	1.10	1.00	0.94	0.91	0.87	0.83

*P*-value								

Wavelength		0.562	0.629	0.744	0.844	0.900	0.938	0.976
Block		0.001	0.0004	0.0001	<0.0001	<0.0001	<0.0001	<0.0001

1 Number of experimental units. Experimental unit = 1 incubator containing 238, 208, 212 and 200 eggs in trial 1, 2, 3 and 4, respectively.

**Table 4:** Effects of the application of colored photoperiodic light during incubation on the spread of hatch (hours interval between specific percentage of hatch) in total hatched Ross 308 broilers.

		% of chicks hatch			
Wavelength	n^1^	5%-95%	10%-90%	25%-75%	50%-75%
Dark	7	23.6	17.9	9.1	4.5
Blue	8	26.3	20.0	10.2	5.0
Red	8	24.8	19.0	9.7	4.7
White	8	24.8	18.7	9.5	4.7
SEM		0.94	0.76	0.41	0.19

*P*-value					

Wavelength		0.319	0.319	0.323	0.321
Block		0.348	0.429	0.490	0.431

1 Number of experimental units. Experimental unit = 1 incubator containing 238, 208, 212 and 200 eggs in trial 1, 2, 3 and 4, respectively.

Counting the number of chicks hatched within certain time intervals and calculating the average incubation time is a common method used for monitoring hatch time (Özkan et al., 2012; Zhang et al., 2016; Sabuncuoglu et al., 2018). However, the information on hatch time distribution within hatching process cannot be drawn by simply comparing the average incubation time. An analysis of the spread of hatch using a nonlinear regression model can provide more information on the distribution of hatch time. Hannah et al. (2020) found a synchronized HW for Lohmann Brown embryos when illuminated with white LED light during incubation. The result from the current study indicated that HW of broiler Ross strain was not synchronized when illuminated with 12 h d^−1^ of light regardless of the colors of light. Differences in HW responses to light stimulation between strains might be related to genetic selection for growth and production.

### Hatchability and Chick Quality

No differences in hatchability of fertile eggs, hatch weight and embryo mortality were found among lighting treatments in the current study (Table 5). Our results agreed with the results reported by Rozenboim et al. (2003) in turkey, Shafey (2004) in layers and Sabuncuoglu et al. (2018) in Japanese quail. Similarly, no difference in hatchability was found among different photoperiods when illuminated with full spectrum fluorescent light on Cobb broiler hatching eggs (Archer et al., 2009; Archer and Mench, 2014a). Zhang et al. (2012, 2016) reported that providing continuous green light (560 nm) during incubation does not affect hatchability, hatch weight, or embryo mortality. However, the effects of incubation lighting illumination on hatchability and embryo mortality were not consistent among studies. Illuminating lighter brown color broiler hatching eggs with excessive light intensity (1430–2080 lux) had negative effects on hatchability and increased embryo mortality as the amount of light that passed through the eggshell may above the optimal level (Shafey et al., 2005). However, Archer et al. (2017) found White Leghorn, broiler and Pekin duck hatching eggs exposed to LED light with white and red bulbs at 250 lux for 12 h d^−1^ had an improvement in hatchability. The lower percentage of early embryo mortality in White Leghorn, broiler and Pekin duck is a possible explanation for higher hatchability when illuminated with LED light. They concluded that hatchability was affected by the combination of white and red light. Light spectrum also plays an important role in affecting hatching performance. Hluchý et al. (2012) reported that a 3 to 4% increase of hatchability for Ross broiler eggs with white light as compared to red and blue light. But only 540 eggs were used in their study (108 eggs per treatment) and the number of eggs may not meet the requirement of comparing hatchability among treatments. The mechanism for this improvement in hatchability and embryo mortality is not clear. The hatchability of fertile eggs was not statistically improved (*P*-value: 0.533) by the exposure to a photoperiod during incubation in the current study. However, hatchability of fertile eggs was numerically higher for blue (89.18%), red (89.33%) and white light (89.02%) treatments compared to dark (87.71%). Differences in hatchability and embryo mortality between those studies and the current study could be influenced by several factors or their combinations, including the type of light source, the composition of light spectrum, strain of bird, breeder age, and eggshell characteristics such as thickness and pigment deposition.

**Table 5:** Effects of the application of colored photoperiodic light during incubation on broiler hatching performance including set egg weight (g egg^−1^), egg weight loss (%) during the first 18 d, fertility (%), hatchability of fertile eggs (%), chick hatch weight (g bird^−1^), early, middle, and late embryo mortality rate (%) in Ross 308 broiler hatching eggs.

Wavelength	n^1^	Egg weight	Egg weight loss^2^	Fertility^3^	Hatchability of fertile^4^	Hatch weight	Early dead^5^	Middle dead^6^	Late dead^7^
Dark	7	64.5	12.67	93.36	87.71	45.2	7.32	0.63	4.32
Blue	8	64.6	11.96	93.81	89.18	45.2	6.73	0.37	3.84
Red	8	64.2	12.40	93.93	89.33	44.6	5.62	0.62	4.43
White	8	64.5	12.05	94.02	89.02	45.3	6.61	0.94	3.43
SEM		0.18	0.320	0.560	0.801	0.26	0.668	0.247	0.736

ANOVA *P*-value									

Wavelength	0.457	0.428	0.8623	0.533	0.248	0.392	0.464	0.766	
Block	<0.0001	0.108	<0.0001	<0.0001	<0.0001	<0.0001	0.373	0.001	

1 Number of experimental units. Experimental unit = 1 incubator containing 238, 208, 212 and 200 eggs in trial 1, 2, 3 and 4, respectively.

2 Egg weight loss percent = (d 0 egg weight–d 18 egg weight)/d 0 egg weight × 100.

3 Fertility = (number of fertile eggs/number of eggs set) × 100.

4 Hatchability of fertile = (number of eggs hatched/number of fertile eggs set) × 100.

5 Early mortality = (number of dead embryos between 1 and 7 d of incubation/number of fertile eggs set) × 100.

6 Middle mortality = (number of dead embryos between 8 and 14 d of incubation/number of fertile eggs set) × 100.

7 Late mortality = (number of dead embryos between 15 d of incubation to external pipping/number of fertile eggs set) × 100.

In the current study, no differences in chick length, average navel scores or the percentage of navel scores of 1 (completely closed and clean) were found among treatments (Table 6). Some previous studies also reported that no effects on chick weight or chick quality at hatch when providing light illumination during incubation (Zhang et al., 2012; Tong et al., 2018). Chick quality, including appearance, activity, characteristics of eye, leg, navel closure condition and other parameters, was not affected by use of green light during the setter phase for Ross broilers (Tong et al., 2018). Several studies find improved navel condition at hatch when incubated under a lighting program. A photoperiod of 12L:12D decreased the percentage of chicks with unhealed navels in comparison to those incubated under dark (Huth and Archer, 2015; Archer, 2017; Archer et al., 2017). Their findings suggested that accelerated embryo development with photostimulation during incubation may result in improved maturation of the navel. However, this was not the case in the current experiment as incubating broiler hatching eggs with blue, white or red light for 12 h d^−1^ affect embryo development or newly hatched chick quality.

**Table 6:** Effects of the application of colored photoperiodic light during incubation on chick length (mm), average navel score, and percent of score 1 (%) in Ross 308 broiler chicks at hatch.

		Chick length		Average navel score		Percentage of score 1	
Wavelength	n^1^	Female	Male	Female	Male	Female	Male
Dark	7	18.8	18.8	1.8	1.6	29.36	39.03
Blue	8	18.9	18.9	1.8	1.7	28.38	36.57
Red	8	18.8	18.7	1.7	1.7	30.92	36.62
White	8	18.9	18.8	1.8	1.7	27.21	36.31
SEM		0.05	0.05	0.03	0.03	2.510	2.510

*P*-value		Chick length		Average navel score		Percentage of score 1	

Wavelength		0.143		0.728		0.767	
Gender		0.100		<0.0001		<0.0001	
W × G		0.933		0.562		0.871	
Block		<0.0001		<0.0001		<0.0001	

1 Number of experimental units. Experimental unit = 40 birds per gender hatched from each incubator.

No differences in yolk-free body weight, relative yolk-free body weight or relative spleen were found among treatments (Table 7). This result was consistent with previous reports (Fairchild and Christensen, 2000; Özkan et al., 2012). Relative bursa of Fabricius weight was affected by the two-way interaction between wavelength and gender (Table 7). Compared to those incubated under dark, male chicks illuminated with blue light had lower relative bursa of Fabricius weight. No differences in the relative bursa of Fabricius weight in female day-old chicks were found among lighting treatments. The result indicated that the response in bursa of Fabricius weight of neonatal chicks to photostimulation depended on gender, which has not been studied or reported before. Bursa of Fabricius is the primary immune organ for the development and maturation of B lymphocyte in avian species. The relative bursa of Fabricius weight can be affected by diet, environmental stress or photostimulation (Xie et al., 2008; Quinteiro-Filho et al., 2010; Li et al., 2015). Li et al. (2015) reported that providing green light during the grow-out period promoted melatonin secretion and its secretion correlated with B lymphocyte proliferation in bursa of Fabricius in broilers. The different results between their study and ours could be the age of animals (2 wk broilers vs. embryo) or the light characteristics (light spectra and intensity) utilized by embryos. However, the mechanism of light spectra, in combination with photoperiods, affecting the structure and function of the primary immune organ of chicken embryos remains unclear. Immunohistochemcial staining for proliferating cell nuclear antigen can be investigated and provide a better understanding in lymphoid organ development with photostimulation.

**Table 7:** Effects of the application of colored photoperiodic light during incubation on yolk-free body weight (YFBW) (g), relative YFBW (%), relative weight (g kg^−1^) of spleen, bursa of Fabricius, and liver in Ross 308 broiler chicks at hatch.

Wavelength	Gender	n^1^	Yolk-free body weight	Relative YFBW	Relative spleen weight	Relative bursa of Fabricius weight	Relative liver weight
Dark	Female	7	37.9	86.89	0.52	1.28^c^	38.59
	Male	7	38.8	86.69	0.57	1.53^a^	36.09
Blue	Female	8	39.6	87.69	0.60	1.44abc	38.60
	Male	8	39.2	86.50	0.54	1.31^bc^	36.07
Red	Female	8	38.6	88.53	0.57	1.39abc	39.61
	Male	8	38.9	87.07	0.55	1.46^ab^	37.44
White	Female	8	38.7	87.31	0.55	1.28^c^	36.21
	Male	8	39.1	87.68	0.58	1.49^ab^	35.38
SEM			0.66	0.600	0.025	0.065	0.943

ANOVA *P*-value							

Wavelength (W)			0.411	0.326	0.754	0.861	0.054
Gender (G)			0.470	0.134	0.918	0.029	0.006
W × G			0.821	0.335	0.212	0.014	0.786
Block			0.0002	<0.0001	0.009	0.0003	0.007

1 Number of experimental units. Experimental unit = 40 birds per gender hatched from each incubator.

a,b,c Means within a column with different letters differ significantly according to Tukey-Kramer test (α = 0.05).

The relative liver weight of chicks hatched under red LED light tended to be higher than those under white light (Table 7). Increased embryo weight at ED 15, 18 and 21, and accelerated liver development at ED 21 in Arbor Acres broiler embryos may be related to higher plasma melatonin level and expression of melatonin receptor 1c in liver when illuminated with continuous LED green light (Wang et al., 2014). However, neither liver nor heart weight differed between dark and photo-stimulation groups in turkey poults when illuminated with incandescent light for 12 h d^−1^ (Fairchild and Christensen, 2000) and Ross 308 broilers exposed to cool white fluorescent light for 16 h d^−1^ (Özkan et al., 2012). Therefore, the difference between findings on relative liver weight may relate to different strains or light regimes. Relative liver weight showed an increased trend (*P* = 0.054) in the current study, which could have been due to increased gluconeogenesis and increased need for mobilization of glucose during late embryogenesis until hatch. Maatjens et al. (2016) reported an increase in liver weight of broilers from internal pipping to hatch when incubated at a lower temperature than optimum during the last week of incubation. In combination with the lower air cell temperature found in embryos incubated with red light illumination, the differences in liver development may be confounded by embryo temperature. A previous study found higher gene expression of peroxisome proliferator activated receptor-γ coactivator 1 α (PGC-1α) when chicken embryos were incubated at 35°C when compared with the 38°C treatment group (Walter and Seebacher, 2007). Their findings suggested that lower incubation temperature induced PGC-1α gene expression and activated gluconeogenesis in chicken embryos. Wu et al. (2001) reported that illuminated chicken embryos with 12 mW cm^−2^ light had a 220% increase in embryo movement compared to 1 mW cm^−2^ light stimulation. The increase in embryo movement with light stimulation may have a higher requirement for glucose as the energy source. Our results indicated that chicks incubated under red light for 12 h d^−1^ may have increased gluconeogenesis in the liver and the effects of light spectra on embryo movement and liver gluconeogenesis need to be investigated in future research.

In conclusion, the results of this study demonstrate that illumination with different colors of LED light for 12 h d^−1^ throughout incubation did not affect hatchability, embryo mortality, spread of hatch or day-old chick quality. The higher relative liver weight found in the red light stimulation treatment suggests that photostimulation during incubation may affect embryo activity and the requirement of energy. The effects of photostimulation on posthatch growth and physiology need to be investigated in the future.
